# Bedside Neuromodulation of Persistent Pain and Allodynia with Caloric Vestibular Stimulation

**DOI:** 10.3390/biomedicines12102365

**Published:** 2024-10-16

**Authors:** Trung T. Ngo, Wendy N. Barsdell, Phillip C. F. Law, Carolyn A. Arnold, Michael J. Chou, Andrew K. Nunn, Douglas J. Brown, Paul B. Fitzgerald, Stephen J. Gibson, Steven M. Miller

**Affiliations:** 1 RECOVER Injury Research Centre, The University of Queensland and Surgical, Treatment & Rehabilitation Service (STARS), Herston, Brisbane, QLD 4029, Australia; 2 Adelaide Neuropsychology, Adelaide, SA 5000, Australia; wendynisca@protonmail.com; 3 Monash Biomedicine Discovery Institute, Department of Physiology, Monash University, Melbourne, VIC 3800, Australia; phillip.law@monash.edu; 4 Caulfield Pain Management & Research Centre, Caulfield Hospital, The Alfred Health, Melbourne, VIC 3162, Australia; c.arnold@alfred.org.au (C.A.A.); profstephengibson@outlook.com (S.J.G.); 5 Department of Anaesthesia & Perioperative Medicine, Monash University, Melbourne, VIC 3004, Australia; 6 Amputee Clinic, Caulfield Hospital, Melbourne, VIC 3162, Australia; m.chou@alfred.org.au (M.J.C.); andrew.nunn@monash.edu (A.K.N.); 7 Victorian Spinal Cord Service, Austin Health, Melbourne, VIC 3084, Australia; 8 Department of Electrical & Computer Systems Engineering, Monash University, Melbourne, VIC 3800, Australia; 9 Spinal Research Institute, Austin Health, Melbourne, VIC 3084, Australia; doug.brown@thesri.org; 10 School of Medicine and Psychology, Australian National University, Acton, ACT 2600, Australia; paul.fitzgerald@anu.edu.au; 11 Monash Alfred Psychiatry Research Centre, Central Clinical School, The Alfred Hospital, Monash University, Melbourne, VIC 3004, Australia; 12 National Ageing Research Institute, Melbourne, VIC 3050, Australia

**Keywords:** vestibular neuromodulation, non-invasive brain stimulation, persistent pain, allodynia, caloric vestibular stimulation, complex regional pain syndrome, phantom limb pain, spinal cord injury pain

## Abstract

Background: Caloric vestibular stimulation (CVS) is a well-established neurological diagnostic technique that also induces many phenomenological modulations, including reductions in phantom limb pain (PLP), spinal cord injury pain (SCIP), and central post-stroke pain. Objective: We aimed to assess in a variety of persistent pain (PP) conditions (i) short-term pain modulation by CVS relative to a forehead ice pack cold-arousal control procedure and (ii) the duration and repeatability of CVS modulations. The tolerability of CVS was also assessed and has been reported separately. Methods: We conducted a convenience-based non-randomised single-blinded placebo-controlled study. Thirty-eight PP patients were assessed (PLP, *n* = 8; SCIP, *n* = 12; complex regional pain syndrome, CRPS, *n* = 14; non-specific PP, *n* = 4). Patients underwent 1–3 separate-day sessions of iced-water right-ear CVS. All but four also underwent the ice pack procedure. Analyses used patient-reported numerical rating scale pain intensity (NRS-PI) scores for pain and allodynia. Results: Across all groups, NRS-PI for pain was significantly lower within 30 min post-CVS than post-ice pack (*p* < 0.01). Average reductions were 24.8% (CVS) and 6.4% (ice pack). CRPS appeared most responsive to CVS, while PLP and SCIP responses were less than expected from previous reports. The strongest CVS pain reductions lasted hours to over three weeks. CVS also induced substantial reductions in allodynia in three of nine allodynic CRPS patients, lasting 24 h to 1 month. As reported elsewhere, only one patient experienced emesis and CVS was widely rated by patients as a tolerable PP management intervention. Conclusions: Although these results require interpretative caution, CVS was found to modulate pain relative to an ice pack control. CVS also modulated allodynia in some cases. CVS should be examined for pain management efficacy using randomised controlled trials.

## 1. Background

Persistent pain (PP) is an increasingly problematic area of healthcare, costing billions of dollars each year [1,2,3,4]. For example, lower back pain is the leading cause of years lived with disability in the majority of countries worldwide, ahead of heart disease, stroke and diabetes [5,6]. Management of PP commonly involves analgesic and anti-neuropathic pain medication, surgery, targeted biomedical interventional techniques, and multidisciplinary pain programs [7,8,9]. PP is frequently refractory to treatment, causing a vicious cycle of pain, deconditioning, social isolation, anxiety, depression, unemployment, and, in turn, worsening pain [10,11].

Risks and harms associated with oral pharmacotherapy for PP, particularly opiate-based preparations, are now widely publicised [12,13,14]. Interventional analgesic techniques for PP include ketamine infusions, targeted injections, radiofrequency denervation and implantable neuromodulation devices such as spinal cord and peripheral nerve stimulators [15]. Although there is some evidence for their efficacy, these techniques are invasive, frequently fail, can cause morbidity and often require repeated administration. Furthermore, the implantable devices and interventions requiring hospitalisation are expensive. Interest has emerged in non-invasive brain stimulation treatments for PP, such as repetitive transcranial magnetic stimulation (rTMS) and transcranial direct current stimulation (tDCS) [16,17]. While safe, these techniques require specialised equipment and can be inconvenient (e.g., daily attendance for weeks) and expensive. Moreover, their efficacy for PP remains to be established [18].

**Figure 1:**
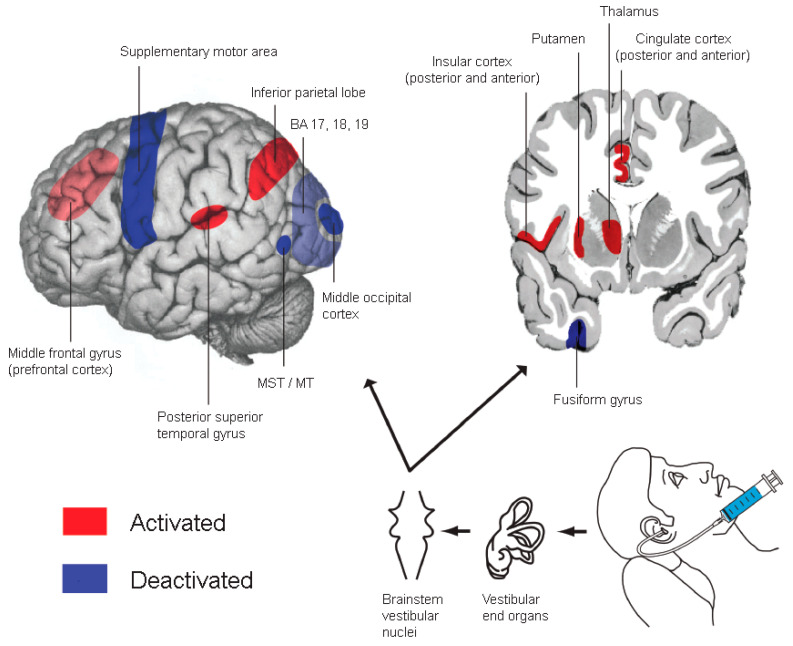
Brain activity associated with caloric vestibular stimulation (CVS). Stronger and better-replicated evidence of CVS-induced brain activation and deactivation is indicated by the darker red and blue tones, respectively. CVS activates—via vestibular pathways (indicated by black arrows)—contralateral structures including the anterior cingulate cortex, insular cortex, putamen in basal ganglia and various temporoparietal areas. Several of these brain regions have been implicated in pain processing [33,34,35,36,37].

Caloric vestibular stimulation (CVS) is a non-invasive neuromodulation technique that is safe, inexpensive and simple to administer by medical or other trained personnel [19,20,21,22]. Bedside CVS involves gentle irrigation of cold water into the external ear canal while the patient is supine with their head angled forward 30° from horizontal [19]. The cold water causes endolymphatic fluid movement in the horizontal semicircular canal, activating the vestibular nerve, brainstem nuclei and predominantly contralateral subcortical and cortical structures (Figure 1; ref. [23,24]). Subjects experience vertigo and an examiner observes horizontal nystagmic eye movements for several minutes (both of which provide evidence of successful stimulation). Bedside CVS is used by neurologists in intensive care units to aid the diagnosis of brain death in comatose patients (with failure to elicit nystagmus suggestive of brain death) [25]. More complicated CVS administration protocols, with detailed interpretations of induced eye movement patterns, have been used by neurologists for decades to diagnose vestibular dysfunction [26].

CVS also induces phenomenological modulations in neurological disorders (e.g., temporarily reversing post-stroke neglect, anosognosia and somatoparaphrenia) and psychiatric disorders (e.g., reducing refractory mania, improving insight in schizophrenia) [19,20,21,22,27]. CVS in healthy subjects modulates a wide range of cognitive and affective functions (e.g., memory, decision-making, mood, bodily representation) [19,20,21,22,27]. These clinical and cognitive modulations raise the prospect of using CVS as a neuromodulation treatment [19,20,21,22,28]. Moreover, neuroimaging studies show that CVS activates structures implicated in various clinical disorders such as the anterior cingulate cortex (ACC), insular cortex (IC), putamen in basal ganglia and temporoparietal regions (Figure 1; ref. [23,24]), further supporting proposals for potential CVS therapeutic efficacy. Examining the therapeutic efficacy of various vestibular stimulation techniques has indeed commenced in neurological contexts [21,22,28,29,30,31,32].

The ACC, IC and putamen are implicated in pain processing and PP states [33,34,35,36,37] and there are reports of pain modulation by CVS. For example, migraines have been reduced by CVS, with effects lasting minutes to days ([38]; see also [31]). Furthermore, an experimental CVS modulation study of phantom limb phenomena in spinal cord injury reported that spinal cord injury pain (SCIP) was greatly relieved in two of four patients [39]. In a related study of amputees, 10 of 10 patients with a painful phantom had pain completely relieved by CVS, with the effect lasting minutes to hours [40]. Analgesic properties of CVS were reported for cases of central post-stroke pain (CPSP, ref. [41,42,43]) and central pain of spinal origin [44]. In two cases of CPSP, CVS also reduced allodynia—pain from a usually non-painful stimulus—with long-lasting reductions. In a CPSP case series of nine patients [42], four had an excellent CVS analgesic response for days to weeks, three had some pain reduction (though not sustained in two and CVS not tolerated in one), and two did not respond at all. Sham CVS with body-temperature water and a cold-arousal control involving an ice pack applied to the ear lobe did not reduce pain or allodynia. CVS analgesic responses were often repeatable. Finally, CVS in healthy subjects has been shown to increase experimental pain thresholds [45,46].

On the basis of reported CVS-induced modulation of pain and allodynia, neuroimaging findings of CVS-induced activation (e.g., ACC, IC, putamen)—and the relative safety, cost-effectiveness and simple administration of the CVS technique—we examined across a variety of common PP conditions (i) short-term pain modulation by CVS relative to a forehead ice pack cold-arousal control procedure and (ii) the duration and repeatability of CVS modulations. We also examined the tolerability of CVS for potential clinical translation and those data were reported elsewhere [47]. We focused on phantom limb pain (PLP), SCIP, complex regional pain syndrome (CRPS) types I and II, and non-specific persistent pain (NPP), given the relative lack of detailed data available for CVS modulation effects in such conditions compared with CPSP. We performed a convenience-based non-randomised study with a single-blinded control for short-term pain modulation effects. Acknowledging the limitations associated with this study design (see Discussion), we aimed to assess if there were pain and allodynia modulations from CVS that warranted proceeding to a formal randomised controlled clinical trial.

## 2. Methods

### 2.1. Participant Recruitment

Recruitment and testing took place at Caulfield Pain Management & Research Centre, Royal Talbot Rehabilitation Centre and Caulfield Hospital’s Amputee unit. Eligibility criteria were age between 18–65 years with (i) PP at or below deafferentation level for PLP and SCIP, (ii) PP in CRPS as diagnosed by a specialist pain physician (note not all subjects may have met Budapest criteria [48] for CRPS at the time of testing) and (iii) musculoskeletal PP for NPP patients. Suitability to participate was confirmed by the patient’s attending medical specialist and author SMM. A semi-structured clinical interview obtained history, diagnosis and treatment information. Exclusion criteria were (i) epilepsy or any brain disorder, (ii) ear disease within the past five years, (iii) pregnancy, (iv) cardiac or respiratory disease (unless cleared by treating specialist) and (v) alcohol or substance dependence or psychiatric comorbidity (except depression). The absence of these conditions was confirmed via medical records and clinical interviews. A total of 38 patients participated in the study (19 male; mean age = 45.6 years; PLP = 8, CRPS = 14, SCIP = 12 and NPP = 4). Participants provided written, informed consent, in accordance with a protocol approved by the Alfred Health Human Research Ethics Committee (145/07), Austin Health Human Research Ethics Committee (H2008/02933) and Monash University Human Research Ethics Committee (CF08/1427-2008000718). All procedures satisfied the standards of the Declaration of Helsinki (1975). This study was retrospectively registered on the Australian New Zealand Clinical Trials Registry (ACTRN12622000661774) on 5/5/22. Handedness was assessed with the Edinburgh Handedness Inventory [49] (for PLP patients, it applied to the period before amputation). Table 1 presents detailed descriptive patient information.

### 2.2. Intervention Procedures

CVS and ice packs were administered on a non-randomised, convenience (patient-availability) basis, in a single testing session or for up to three separate sessions, with CVS never repeated on the same day. The number of days separating repeat interventions varied according to patient availability. Thirty-four patients underwent both CVS and ice pack intervention and four underwent only CVS. The ice pack was often administered on the same day as the initial CVS (CVS#1), with a minimum period of 30 min before CVS occurred. CVS was never administered before ice pack on the same day. There was no formal randomisation or decision algorithm regarding whether or not a patient received an ice pack prior to CVS#1. Intervention timing and order are indicated in Table 1, together with the number of days separating sessions.

CVS was administered with patients seated or lying with their heads angled forward 30° from horizontal. Up to 50 mls of iced water (0–4 °C) was slowly irrigated into the right external ear canal using a syringe with attached plastic cannula tubing (with the needle removed) that was situated close to, but not touching, the tympanic membrane. Only right-ear CVS was applied to avoid a theoretical risk of worsening comorbid depression ([22], though see [50]). To ensure maximal stimulation, irrigation continued for 5–10 s after nystagmus onset. Nystagmus and vertigo were elicited in all patients and persisted for 2–5 min. The ice pack was applied to the patient’s forehead until cold-related discomfort was unbearable. The ice pack never induced nystagmus or vertigo. Patients were informed that the effects of ‘thermal stimulation’ on PP were being investigated and hence were blind to CVS being the intervention of interest (though the success of blinding was not assessed).

### 2.3. Outcome Measures

Outcome variables comprised subjective ratings of PP and allodynia, as well as narrative reports of pain. In addition, qualitative reports of sensations, perceptions and beliefs concerning pain-affected limbs were collected and will be the subject of a separate report. Subjective reports of mood were collected as a secondary outcome, given the potential for CVS to modulate mood [22]. Outcome variables were collected at (i) baseline (before any intervention); (ii) after nystagmus settled for CVS and at 10, 20 and 30 min post-intervention; (iii) roughly the same time after ice pack and at 10, 20 and 30 min post-intervention; and (iv) at follow-up (see below). Measures included a numerical rating scale for pain intensity (NRS-PI) with scores from 0 (‘no pain’) to 10 (‘worst pain imaginable’) and a numerical rating scale for mood with scores from 0 (‘worst you have ever felt’) to 10 (‘best you have ever felt’). Follow-up assessments were conducted in person or by telephone 24 h post-intervention. Patients who showed pain or allodynia reductions within the initial 24 h were followed up at approximately weekly intervals for up to four weeks. Follow-up NRS-PI for pain used average pain ratings in the previous 24 h. Dynamic allodynia in CRPS was examined using light finger or tissue-paper strokes and was also rated with NRS-PI in response to the stimulus. Follow-up NRS-PI for allodynia used (i) the same stimulus when the patient was assessed in person or (ii) self-reported general allodynia levels in the previous 24 h when follow-up was conducted by phone. As reported elsewhere [47], 25 of 38 patients also rated their experience of CVS as uncomfortable or painful (including intensity scores), stated whether they experienced nausea or headache (and associated intensity) or other symptoms, and indicated if they would repeat the procedure if it reduced their pain by 50% or more for 1 week or 1 month.

### 2.4. Analyses

Quantitative analyses were conducted on data from the 34 patients who underwent both CVS and the ice pack intervention. Baseline measures were compared using paired sample *t*-tests. A two-by-two (Time: Pre, <30 min post|Intervention: CVS#1, ice pack) repeated-measures MANOVA was performed on numerical pain and mood ratings, collapsed across the four different PP conditions. Not every patient underwent multiple sessions of CVS due to patient inconvenience or scheduling limitations. As such, data from CVS#1 were analysed in each case to maximise power. To compare self-reported allodynia intensity at pre- and post-CVS (<30-min) and pre- and post-ice pack, a two-way repeated-measures ANOVA was performed. When ratings within the 30 min post-intervention differed from the baseline (pre-intervention) rating, the post-intervention rating most different from baseline was used in analyses. CRPS (*n* = 1), PLP (*n* = 3), SCIP (*n* = 2) and NPP (*n* = 1) patients reporting no or almost no pain at baseline were nonetheless included in analyses. For these patients, assessing for reports of delayed pain reductions used average pain ratings in the 24 h prior to CVS as the baseline value.

## 3. Results

### 3.1. Short-Term Intervention Effects on Pain

**Figure 2:**
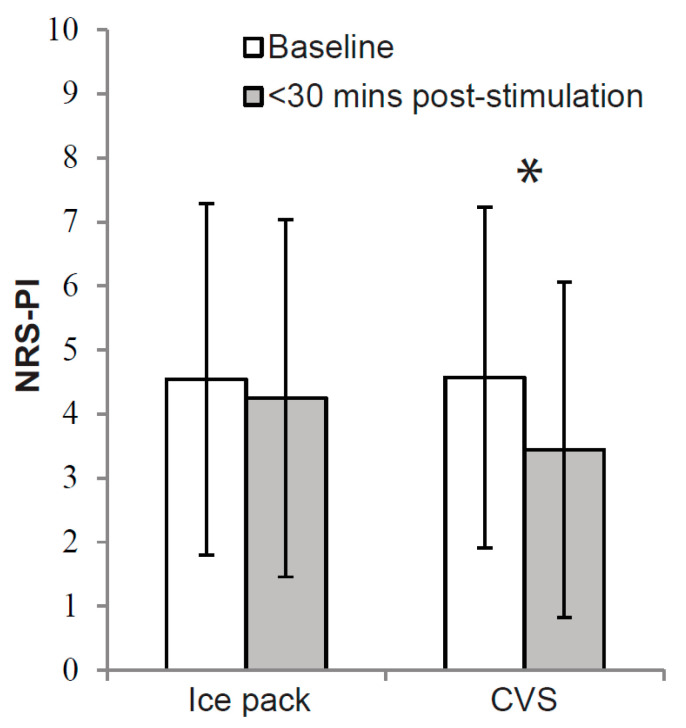
Mean pain intensity at baseline and up to 30 min post-CVS#1 and post-ice pack intervention for all patients who underwent both conditions (N = 34). Results revealed significantly lower pain ratings compared to baseline within 30 min post-CVS than post-ice pack intervention. Error bars designate standard deviations. * Results revealed significantly lower pain ratings compared to baseline within 30 min post-CVS than post-ice pack intervention.

Baseline pain ratings immediately before CVS (*M* = 4.57, *SD* = 2.66) were not significantly different from those before the ice pack intervention (*M* = 4.54, *SD* = 2.75, *t*(33) = 0.10, *p* = 0.920). Baseline mood ratings immediately before CVS (*M* = 6.78, *SD* = 1.49) also did not significantly differ from those before the ice pack intervention (*M* = 6.53, *SD* = 1.35, *t*(33) = 1.55, *p* = 0.130 (two-tailed)). A MANOVA of pain and mood scores showed a significant main effect of time (*F* [2,32] = 6.89, *p* < 0.01) but not intervention (*F* [2,32] = 2.43, *p* = 0.104). There was, however, a significant interaction of time with intervention, suggesting that outcomes across time differed between CVS and the ice pack intervention (*F* [2,32] = 3.99, *p* < 0.05). Univariate tests revealed that pain scores differed significantly across time between CVS and the ice pack intervention (*F* [1,33] = 17.30, *p* < 0.01), but mood scores did not (*F* [1,33] = 1.31, *p* = 0.260). Compared with baseline, pain ratings were significantly lower within 30 min after CVS *(M* = 3.44, *SD* = 2.62) than after the ice pack intervention (*M* = 4.25, *SD* = 2.79, *t*(33) = −3.77, *p* < 0.01 (see Figure 2)). Cohen’s *d* effect size was 0.3 (i.e., small to medium; ref. [51]). More specifically, pain reduced from baseline by an average of 24.8% or 1.13 NRS-PI points (*SD* = 1.67) within 30 min after CVS, compared to 6.4% or 0.29 points (*SD* = 1.21) after the ice pack intervention.

### 3.2. Short-Term or Delayed Intervention Effects on Pain and the Duration, Repeatability and Laterality of Effects

**Figure 3:**
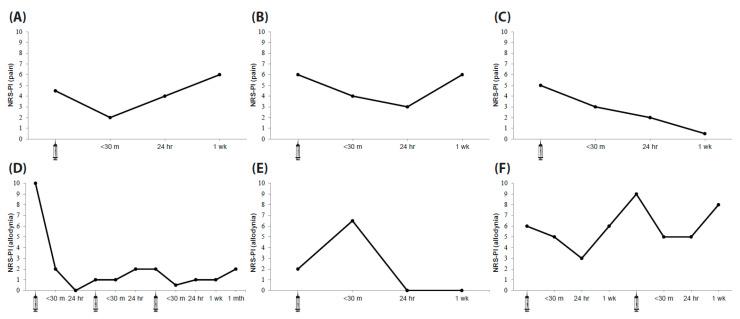
Case illustrations of pain and allodynia modulation by CVS: (**A**) CRPS-12—a case of ≥50% short-term pain reduction following CVS that had returned to baseline by 24 h. (**B**) CRPS-13—a case of ≥30–49% short-term pain reduction following CVS that became a ≥50% reduction by 24 h, returning to baseline by 1 week. (**C**) NPP-1—a case of ≥30–49% short-term pain reduction following CVS that became a ≥50% reduction by 24 h, still evident after 1 week. (**D**) CRPS-2—a striking case of ≥50% short-term allodynia reduction following CVS, completely ameliorated allodynia by 24 h, two further CVS sessions on consecutive days keeping allodynia at low levels, repeat reduction evident after CVS#3 and allodynia reported to be still low (2/10) a month later. (**E**) CRPS-6—a case of a short-lived increase in allodynia following CVS with amelioration of allodynia by 24 h and allodynia still absent after 1 week. (**F**) CRPS-11—a case of <30% allodynia reduction following CVS that became a ≥50% allodynia reduction by 24 h, returning to baseline by 1 week and a repeatable reduction after a second CVS session.

The proportion of short-term or delayed pain responses following CVS according to clinical group is presented in Table 2. Although not quantitative in nature, noteworthy narrative reports for pain reductions included reference to pain being ‘the best it had ever been’ (CRPS-1, CRPS-11). In some cases, narrative reports for pain suggested a degree of CVS modulation, even though numerical ratings did not change. The duration of pain reduction following CVS varied from minutes to over three weeks. Three cases of ≥50% pain reductions are depicted in Figure 3A–C. Data on the duration of CVS effects were in some cases limited by CVS being repeated on the subsequent day.

Data on the repeatability of CVS pain reductions were limited by the large variability in times between CVS#1 and repeat CVS sessions and because not all patients underwent repeat CVS. From the available data, however, the repeat CVS pain modulation effects varied. Patients experienced pain reductions regardless of injury laterality; however, longer pain reductions were more common when pain was contralateral to the activated (left) hemisphere. A few patients reported short-term increases in pain from either CVS or the ice pack intervention.

### 3.3. Short-Term Intervention Effects on Allodynia

In nine allodynic CRPS patients (cases indicated in Table 1), subjective ratings of stimulus-evoked allodynia intensity before CVS (*M* = 6.22, *SD* = 2.44) were not significantly different from those before the ice pack intervention (*M* = 5.78, *SD* = 1.86, *t*(8) = −0.35, *p* = 0.736). When self-reported stimulus-evoked allodynia intensity was compared between pre- and post-CVS (*M* = 5.44, *SD* = 1.94) and post-ice pack intervention (*M* = 6.33, *SD* = 2.18), no main effect of time or intervention was found (*F* [1,8] = 0.05, *p* = 0.836 and *F* [1,8] = 0.61, *p* = 0.811, respectively). The interaction of intervention and time was also not significant (*F* [1,8] = 2.21, *p* = 0.176). Despite no statistically significant effect on allodynia in the post-CVS 30 min period (i.e., the period for which quantitative statistical analysis was able to be conducted), allodynia had substantially reduced by 24 h following CVS in three CRPS patients, including one with an immediate effect.

### 3.4. Short-Term or Delayed Intervention Effects on Allodynia and the Duration, Repeatability and Laterality of Effects

The proportion of allodynic short-term or delayed responses in CRPS patients with allodynia is presented in Table 2. Noteworthy narrative reports for CVS allodynia modulations included reference to being able to wear long-sleeve garments for the first time since injury (CRPS-2) and being able to tolerate being in the wind (CRPS-6). In some cases, narrative reports for allodynia suggested a degree of CVS modulation, even though numerical ratings did not change. The duration of allodynia reductions ranged from hours to 1 month. Three cases of ≥50% allodynia reductions are depicted in Figure 3D–F, including one with a short-term increase in allodynia post-CVS. Pain and allodynia reductions were usually concordant but were sometimes dissociated. The repeatability of CVS allodynia modulations also varied. Patients experienced allodynia reductions regardless of injury laterality. A variety of sensation changes were reported in CRPS, PLP and SCIP groups and these will be the subject of a separate report.

### 3.5. Tolerability of CVS

**Figure 4:**
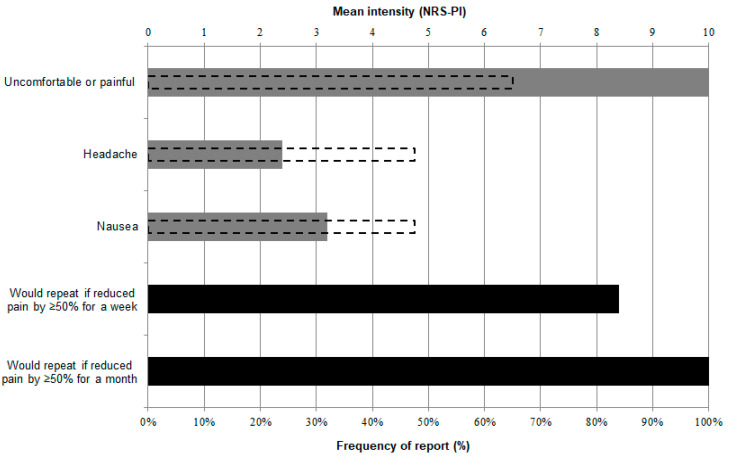
Tolerability and side effects of CVS and patient willingness to repeat the intervention if it reduced their pain by 50% or more for one week or one month (figure reprinted from [47]). Solid bars indicate the percentage of patients (*n* = 25 with formal tolerability data available), while dotted lines indicate the reported intensity of CVS-induced discomfort/pain and side effects. The vast majority of patients reported that despite finding the procedure uncomfortable or painful or experiencing side effects, they were willing to repeat the intervention if it helped their pain.

Tolerability data from this study were the subject of a separate report [47] and the findings are described in Figure 4.

## 4. Discussion

### 4.1. CVS Modulation of PP and Allodynia

We observed a single session of right-ear CVS to induce an approximately 25% reduction in subjective PP within 30 min following stimulation across four PP conditions. This finding was a statistically significant pain reduction relative to an ice pack cold-arousal control procedure, which by contrast induced a four-fold lower reduction in pain. Mood ratings did not change significantly following CVS, indicating that the significant pain reductions cannot be explained by CVS effects on mood. Our findings confirm reports in the literature that activation of the vestibular system modulates pain processing [31,38,39,40,41,42,43,44,45,46].

In the CRPS group, nearly all patients reported short-term or delayed pain reductions and more than half of these were of ≥30–49% or ≥50% magnitude. Additionally, this group exhibited some noteworthy cases of either short-term or delayed allodynia reductions of ≥30–49% or ≥50% (including cases of allodynia reductions lasting 19 days and 1 month). While ≥30–49% is considered a moderate clinically important difference and ≥50% is considered a substantial clinically important difference [52], the percentages reported here are associated with either short-term or delayed modulations, and clinical importance must take into account analgesic duration and repeatability. We observed durations of pain reductions to vary from minutes to weeks and allodynia reductions from hours to 1 month. Pain reductions following repeat CVS were variable. In our view, the most striking finding we observed was substantial pain and/or allodynia reduction in a large number of CRPS cases, with accompanying narrative comments suggestive of potential clinical translation.

However, several aspects of this study limit claims for clinical implications. As mentioned, the focus was on short-term modulation, and the data were limited for the duration and repeatability of modulation effects. Even when CVS was repeated, it was never more than three sessions in total and the time between CVS sessions was sometimes long (cf. [50]). Moreover, the study protocol was convenience-based, involved variability and non-random allocation of intervention order, and was not double-blinded. Indeed, even the single-blinding element of our study may not be valid because although patients were informed that ‘thermal stimulation’ was being investigated, there was no specific assessment performed to verify the efficacy of this single-blinding. All of these design and methodology factors create multiple sources of potential bias and necessitate cautious interpretation of the findings. Placebo effects in pain research are substantial [53] and we cannot rule out such effects given the limitations of this study’s design and methodology. Another limitation was the lack of a pain diary to better track longer CVS responses and a lack of standardised outcome measures such as the Brief Pain Inventory (cf. [50]). In addition, a formal diagnosis of CRPS according to Budapest criteria [48] did not occur; hence, not all subjects designated as having CRPS necessarily met such criteria at the time of testing. Notwithstanding the aforementioned requirement for substantial interpretive caution, the present data contribute to existing case series data suggesting that CVS warrants further investigation using well-powered double-blind randomised controlled trials (RCTs).

We did not, however, observe ‘great relief’ of pain following CVS in half of our SCIP patients, as was observed (albeit in passing) by others [39]. In a separate case study of cervical transverse myelitis, CVS (but not control procedures) reduced neck and right upper limb pain for 10 days, with pain at its lowest in years [44]. In our study, we observed few cases of ≥50% SCIP modulation by CVS and fewer short-term reductions in PLP than those observed by others [40]. However, PLP and SCIP are difficult to study due to pain not always being present just before CVS, as was the case for several of our patients. It is possible though that more clinically relevant PLP and SCIP analgesic effects of CVS could be observed with repeated CVS sessions. Additionally, further research may elucidate which PLP and SCIP patients predictably respond to CVS. Interestingly, a recent larger study provides more compelling data for CVS modulation of PLP (N = 34; ref. [54]). Additional features of that study were the use of a homogenous PLP cohort and reported pain reductions despite the use of milder CVS (i.e., water at 30 °C cf. 0–4 °C in the present study).

Reports in the literature suggest that CVS reduces central pain following stroke. In one patient with CPSP, CVS reduced pain (the best pain relief in over a decade), the effect was repeatable, pain remained reduced for 4 weeks and relief was more pronounced for the face/arm than the lower limb [41]. In another CPSP case [41], CVS reduced pain (the best it had been for years), again more so for the face/arm than the leg, with an associated 7-h reduction in allodynia and further reductions from repeat CVS lasting nearly two months. These two CPSP cases were extended to nine [42], with CVS and an ice pack intervention inducing, respectively, mean short-term pain reductions of ~2.5 and ~0.5 points on a 1–10 point scale, with variable durations of CVS pain reduction and two patients not responding at all. Placebo effects were considered unlikely given the poor response to sham CVS or the ice pack intervention, long-lasting analgesic effects, better pain reductions for the face/arm than the lower limb, and poor response to more invasive interventions. In other CPSP cases, CVS reduced pain and allodynia for at least 4 days [43] and reduced mouth/shoulder pain for 3–5 h, which was repeatable ([55]; though see [56]).

We similarly observed highly variable features of CVS modulations, including cases of (i) short-term pain reductions, (ii) longer pain reductions, (iii) delayed pain reductions, (iv) pain non-responders, (v) pain response only at CVS#2 or CVS#3, (vi) short-term allodynia reductions, (vii) longer allodynia reductions, (viii) 24-h delayed allodynia reductions, (ix) allodynia non-responders and (x) variable repeat CVS effects. In a recent case study [50], we also report repeatable, substantially delayed CVS pain and allodynia reductions, evident ~8 days post-stimulation (along with initial short-term reductions). This case involved the use of a daily pain and allodynia diary, was associated with highly clinically important improvements, and illustrates how CVS might obviate the requirement for more expensive, invasive neuromodulation techniques.

### 4.2. Tolerability of CVS as an Intervention for PP

We previously reported, using data from this study, that CVS is a well-tolerated intervention in a PP cohort [47]. This was evidenced by the readiness to repeat the procedure if it improved pain in the vast majority of patients (Figure 4). There was a high willingness to undergo CVS to treat pain despite it being considered uncomfortable or painful by all patients and causing nausea and headache in one-third and one-quarter, respectively ([47]; Figure 4). It is important to note that one patient did exhibit repeated emesis due to CVS and he did not accept, nor was offered, repeat CVS. There are other reported cases of CVS being poorly tolerated, even when pain reduction is induced [42]. Because of such reports, one research group proposed galvanic vestibular stimulation (GVS) as a potential alternative to CVS as a PP treatment [57]. From their study showing the modulation of experimental pain by GVS, the authors extrapolated and raised the possibility of using GVS to manage PP, noting that GVS was well tolerated. In response, we reported CVS tolerability data from the present study and noted that although GVS could indeed be a potential alternative for subjects who do not tolerate CVS, such subjects are not in fact commonly observed in PP cohorts [47].

Two further points are worth discussing in relation to CVS tolerability. First, our tolerability data [47] appear more favourable than those recently reported for CVS in the vestibular diagnostic context [58]. In that study, of 130 patients, 75 (58%) reported side effects including nausea (50%), vomiting (5%) and headaches (12%). The ratings for distress and nausea were generally low though (<3/10), with the exception of 19 (15%) patients whose symptoms led to the discontinuation of testing. However, CVS in this diagnostic setting is just one part of a broader, more onerous investigation suite [58] than single sessions of CVS, as used in the present study. Moreover, tolerability data from a study of CVS used in the diagnostic context may not readily extrapolate to the treatment context, particularly when treating PP. This is because PP patients have often already undergone a range of uncomfortable and invasive treatments. This experience, along with the sustained experience of pain associated with a pain condition needing treatment, may increase the tolerance PP patients exhibit for the discomfort associated with CVS.

Second, and importantly, when assessing the tolerability of any potential new PP treatment, there needs to be a comparison with the discomfort and side effects associated with existing pharmacotherapeutic and interventional PP treatment options [13,14,15], as well as the biological, psychological and social sequelae of otherwise refractory PP [11,12]. Analgesic and antineuropathic pain medications frequently cause side effects lasting substantially longer and being more problematic than transient nausea/headache, and opiates can additionally be associated with dependence, sedation and mortality risk [13,14]. Similarly, ketamine infusions frequently need to be discontinued due to intolerable adverse psychological side effects (or elevated liver enzymes), while invasive PP interventions such as targeted injections, radiofrequency denervations and spinal cord stimulation may involve significant initial discomfort and are not without morbidity risks both during administration and in the longer term [15]. When viewed against this background, and notwithstanding some patients for whom CVS will not be tolerated, the side effect profile we reported for CVS in a persistent pain cohort [47] suggests that the technique, if efficacious, would be considered an acceptable PP intervention.

In addition to a tolerability advantage, there are several other advantages of CVS relative to existing PP interventions and emerging PP brain stimulation methods (discussed more fully in [28]). These include (i) safety, (ii) ease of administration (including wide availability in rural/remote areas and developing countries), (iii) lack of required specialised equipment, (iv) lack of complex administration variables, (v) potential for home-based self-administration (after medical guidance and training), (vi) ready evidence of successful brain stimulation (vertigo, nystagmus) and (vii) negligible expense.

## 5. Future Directions

Further studies are required to assess the therapeutic efficacy of CVS for managing PP. This work will require well-powered RCTs to exclude placebo effects and sources of bias, though further case series can inform RCT planning, particularly given the highly variable CVS modulation effects. The ease with which CVS can be administered at the bedside and with no special training required for medical personnel makes the performing of potentially informative case series by pain physicians and related specialists a viable proposition. Methodological and related issues to consider when evaluating CVS as a potential treatment technique, including the issue of options for control procedures, are discussed elsewhere, as are related vestibular stimulation techniques that have previously been and are currently being assessed for therapeutic efficacy [19,22,28,59].

CVS research in PP can also widen to assess, for example, pain associated with multiple sclerosis, diabetes, HIV and chemotherapy, as well as trigeminal, occipital and post-herpetic neuralgias, all of which can be difficult to treat. The data presented here (see also [50]) argue for an initial focus on pain conditions commonly associated with allodynia. Future research can also examine CVS modulation of allodynia using quantitative sensory testing methods and seek to identify patient-specific predictors of response to CVS for allodynia. Similarly, identifying whether, and why, particular underlying pain diagnoses and diagnostic subtypes preferentially respond to CVS will be important for future research, as will understanding variability in the magnitude, onset and duration of pain and allodynia responses. In relation to underlying diagnosis, it is interesting to note that a recent open-label trial of a single CVS session in refractory fibromyalgia induced significant pain reductions at 7-day follow-up relative to baseline pain scores [60]. For a more detailed discussion of technical and methodological issues relevant to future research on CVS in PP and other clinical conditions, see Fraser and Miller [28].

Mechanisms underlying CVS modulation of pain and allodynia also need elucidating. While it is likely that CVS exerts analgesic effects at least in part via its activation of the IC, ACC and putamen [33,34,35,36,37,41,42,43,44], a recent high-profile mouse model study showed that allodynia is modulated by descending corticospinal tract (CST) neurons previously thought to mediate only motor function [61]. Some allodynia-modulating CST neurons originated in the secondary somatosensory area (S2)—an area activated by CVS [23,24]. Thus, we propose that CVS may modulate allodynia via this newly described descending pathway. Finally, there are two additional mechanistic elements of CVS that require further examination and have relevance to PP. The first is the relevance of modulation of opiodergic activity when activating vestibular processing [62]. The second concerns the vestibular system’s capacity to modulate both mood [19,20,21,22,63] and sleep [21,22,28,64,65,66], given the established association between pain, mood and sleep disorders [67,68,69]. Although we did not in the present study observe significant mood changes following CVS, further studies could examine this issue with more sensitive measures for mood and affective processing.

## 6. Conclusions

We have shown that a single session of cold-water CVS significantly reduces pain relative to a cold-arousal control within 30 min post-intervention. While the magnitude of pain reduction in some groups was modest and there were many non-responders, the CRPS group in particular appeared most responsive to CVS pain modulation and also exhibited some striking cases of CVS allodynia modulation. Though requiring cautious interpretation, the current findings confirm that activation of the vestibular system can modulate pain processing and contribute to data suggesting that CVS warrants examination for clinical efficacy. CVS is also well tolerated in PP patients and if therapeutic efficacy is demonstrated in well-controlled studies, the technique would offer a simple, safe, inexpensive and accessible bedside neuromodulation technique for PP management.

## Figures and Tables

**Table 1:** Description of participants including interventions administered.

Case	Handedness (EHI Score)	Region (Lat., Aetiology, Yrs Since Pain Onset) CRPS Type	Pain Descriptor	Medication	Session: Intervention (Days between Sessions)
CRPS-1	R(100)	Knee(R, In, 9) CRPS II	Aching Crushing	Codeine Opioids Paracetamol Tricyclic AD	1: CVS
CRPS-2 ^A^	R(100)	Hand(R, In, 1) CRPS II	Burning Tight	Paracetamol Tricyclic AD	1: CVS(1) 2: ice pack, CVS(1) 3: ice pack, CVS
CRPS-3 ^A^	R(44.4)	Wrist/thumb(L, T, 2) CRPS II	Aching	Paracetamol Tricyclic AD	1: ice pack, CVS
CRPS-4 ^A^	R(100)	Leg(L, T, 2) CRPS II	Gnawing	Tricyclic AD	1: ice pack, CVS
CRPS-5 ^A^	R(80.95)	Foot(R, In, 10 months) CRPS I	Burning Throbbing	Codeine	1: ice pack, CVS(14) 2: CVS
CRPS-6 ^A^	R(83.33)	Shoulder(In, 3) CRPS I	Aching Burning Dull	Paracetamol Tricyclic AD Gabapentin Ibuprofen	1: ice pack, CVS
CRPS-7	R(+100)	Ankle(L, T, 28) CRPS II	Aching	Opioids Tricyclic AD Benzodiazepine	1: ice pack, CVS
CRPS-8 ^A^	R(+83)	Ankle(L, T, 10 months) CRPS II	Aching	Codeine Opioids Paracetamol SNRI	1: ice pack, CVS(6) 2: CVS(6) 3: CVS
CRPS-9 ^A^	R(+79.16)	Ankle(L, T, 7 months) CRPS I	Burning Throbbing	Nil	1: ice pack, CVS
CRPS-10	R(+67.19)	Foot(R, T, 1) CRPS II	Aching Burning Dull Throbbing	Opioids Tricyclic AD NSAID Pregabalin	1: ice pack, CVS(21) 2: CVS(7) 3: CVS
CRPS-11 ^A^	R(+58.33)	Wrist(R, T, 7 months) CRPS I	Burning Shock-like Throbbing Sharp	Paracetamol Tricyclic AD	1: ice pack, CVS(13) 2: CVS(12) 3: CVS
CRPS-12	R(+100)	Foot(L, In, 7 months) CRPS I	Shooting Sore	Tricyclic AD	1: CVS(108) 2: ice pack
CRPS-13 ^A^	L(−100)	Hand(L, Rand, 2) CRPS I	Aching Dull Stabbing	Paracetamol Tricyclic AD	1: CVS(94) 2: ice pack
CRPS-14	R(+100)	Ankle(L)/Hand(R, 6) CRPS I	Radiating Shock-like Throbbing Sharp	Tricyclic AD	1: CVS(7) 2: ice pack
PLP-1	R(+88.89)	TH(R, T, 7)	Pulsing Shooting	Opioids Paracetamol Pregabalin	1: ice pack, CVS
PLP-2	R(+80)	TF(L, I, 9)	Shooting	Codeine Opioids Paracetamol Benzodiazepine NSAID	1: CVS(1) 2: CVS(1) 3: CVS
PLP-3	R(+85)	TF(L, V, 4)	Shock-like Throbbing	Codeine Opioids Paracetamol	1: ice pack, CVS(21) 2: CVS(1) 3: CVS
PLP-4	R(+83.33)	HD(Bi, T, 29)	Sharp Stabbing	Nil	1: CVS(1) 2: CVS(1) 3: CVS(1300) 4: ice pack
PLP-5	R(+100)	TT(R, T, 5)	Sharp Stabbing	Nil	1: CVS(7) 2: CVS(373) 3: ice pack
PLP-6	L(−74)	TH(R, T, 4)	Cramping Burning Sharp	Nil	1: ice pack, CVS(3) 2: CVS(7) 3: CVS
PLP-7	R(+100)	TT(R)/TR(L, T, 38)	Sharp Cutting	Codeine Gabapentin Paracetamol	1: CVS
PLP-8	R(+100)	Toes(Bi, V, 11)	Burning	Opioids Gabapentin	1: ice pack (7) 2: CVS
SCIP-1	R(+91.67)	Tupper(T, 16)	Burning Shock-like	Pregabalin	1: ice pack, CVS
SCIP-2	R(+91.67)	C5/C6(T, 20)	Throbbing Tight Cramping	Gabapentin	1: ice pack (6) 2: CVS
SCIP-3	R(+75)	T6(T, 3 months)	Throbbing	Opioids Paracetamol Pregabalin	1: CVS(1) 2: ice pack
SCIP-4	R(+80.95)	T6(T, 5)	Sharp Throbbing	Pregabalin	1: ice pack, CVS(6) 2: CVS
SCIP-5	R(+75)	T12(T, 15)	Burning Shock-like	Nil	1: ice pack (37) 2: ice pack (20) 3: CVS
SCIP-6	R(+60)	C5/C6(T, 9)	Dull	Paracetamol Pregabalin	1: ice pack, CVS(30) 2: CVS
SCIP-7	A(+33.33)	C4(T, 8)	Burning Sharp	Opioids Tricyclic AD	1: ice pack, CVS(31) 2: CVS
SCIP-8	R(+91.67)	T12(T, 18.5)	Burning Pulsing Stabbing	Pregabalin	1: ice pack, CVS
SCIP-9	R(+80)	T10(T, 2)	Tight	Pregabalin	1: CVS(137) 2: ice pack
SCIP-10	L(−100)	C4(T, 47)	Dull Shock-like Stingy	Pregabalin	1: CVS
SCIP-11 ^B^	L(−100)	T4(T, 1 month)	Shock-like Pulsing Sharp	Pregabalin	1: ice pack (73) 2: CVS
SCIP-12 ^B^	R(+100)	T6(T, 1.2 months)	Tight Sharp	Paracetamol Tricyclic AD	1: ice pack (7) 2: CVS(7) 3: CVS
NPP-1	A(+8.33)	Groin(L, T, 18)	Aching Dull	n/a	1: ice pack, CVS
NPP-2	R(+100)	Lumbar spine(Bi, In, 5)	Aching Dull	Opioids	1: ice pack, CVS
NPP-3	R(+67)	Lower back(Bi, T, 37)	Aching Dull	Codeine Paracetamol	1: ice pack, CVS(1) 2: CVS(14) 3: CVS
NPP-4	R(+100)	Ankle(R, In, 4)	Throbbing Sharp	Opioids Tricyclic AD	1: CVS(3) 2: ice pack

^A^ Allodynia. ^B^ Inpatient. Condition: CRPS = complex regional pain syndrome; PLP = phantom limb pain; SCIP = spinal cord injury pain; NPP = non-specific persistent pain. Laterality: L = left; R = right; A = ambidextrous; Bi = bilateral. Aetiology: In = injury; T = traumatic; I = infection; Rand = random onset; V = vascular. Region: HD = hip disarticulation; TF = transfemoral; TH = transhumeral; TT = transtibial. Medication: AD = antidepressant; NSAID = non-steroidal anti-inflammatory drug; SNRI = serotonin–norepinephrine reuptake inhibitor; n/a = not available. Stimulation: CVS = caloric vestibular stimulation.

**Table 2:** Proportion of pain and allodynia short-term or delayed responses following CVS according to clinical group.

Degree of Response	CRPS (*n* = 14)	PLP (*n* = 8)	SCIP (*n* = 12)	NPP (*n* = 4)	CRPS Allodynia (*n* = 9)
≥50% reduction	*n* = 4 ^a^	*n* = 2 ^c^	*n* = 2 ^e^	*n* = 1	*n* = 3 ^f^
≥30–49% reduction	*n* = 3	*n* = 2 ^d^	*n* = 1	*n* = 1	*n* = 0
<30% reduction	*n* = 5	*n* = 0	*n* = 3	*n* = 1	*n* = 2
No response	*n* = 2 ^b^	*n* = 4	*n* = 6	*n* = 1	*n* = 4

^a^ One with ≥50% IP response. ^b^ One with no baseline pain or allodynia for ankle CRPS but ≥30–49% reduction in separate knee pain. ^c^ One with delayed response. ^d^ One with delayed response and one reporting that IP was more effective than CVS. ^e^ One with ≥50% IP response and one with ≥30–49% IP response. ^f^ Two with delayed response.

## Data Availability

The datasets used and/or analysed during the current study are available from the corresponding author upon reasonable request.

## Abbreviations

CVS: caloric vestibular stimulation; CRPS: complex regional pain syndrome; NPP: non-specific persistent pain; CPSP: central post-stroke pain; PLP: phantom limb pain; PP: persistent pain; SCIP: spinal cord injury pain; tDCS: transcranial direct current stimulation; rTMS: repetitive transcranial magnetic stimulation; NRS-PI: numerical rating scale pain intensity; ACC: anterior cingulate cortex; IC: insular cortex; RCT: randomised controlled trial.
